# Infectious disease specialists and teamwork strategies worldwide: the World Association against Infection in Orthopedics and Trauma (WAIOT) and SICOT continue to cooperate in fighting musculoskeletal infections

**DOI:** 10.1051/sicotj/2022031

**Published:** 2022-08-15

**Authors:** Carlo L. Romanò, Andreas G. Tsantes, Dimitrios V. Papadopoulos, Hiroyuki Tsuchiya, Thami Benzakour, Joseph Benevenia, Hernán del Sel, Lorenzo Drago, Andreas F. Mavrogenis, World Association against Infection in Orthopedics and Trauma (WAIOT) Study Group on Bone and Joint Infection Definitions

**Affiliations:** 1 Studio Medico Cecca-Romano – Corso Venezia 20121 Milano Italy; 2 Department of Microbiology, Saint Savvas Oncology Hospital 115 22 Athens Greece; 3 Department of Orthopaedics, Geisinger Medical Center Danville PA 17822 USA; 4 Department of Orthopaedic Surgery – Graduate School of Medical Sciences, Kanazawa University 920-0293 Kanazawa Japan; 5 Zerktouni Orthopaedic Clinic 20000 Casablanca Morocco; 6 Department of Orthopaedics, Rutgers New Jersey Medical School Newark NJ 07103 USA; 7 Department of Orthopaedics, British Hospital Buenos Aires C1280 AEB Buenos Aires Argentina; 8 Clinical Microbiology, University of Milan 20122 Milano Italy; 9 First Department of Orthopaedics, National and Kapodistrian University of Athens, School of Medicine 11527 Athens Greece

**Keywords:** Bone and joint infections, WAIOT, SICOT, Training, Orthopaedics, Infectious diseases specialists

## Abstract

Bone and joint infections are associated with a devastating global burden. The successful treatment of these infections requires a multidisciplinary approach between orthopedic surgeons and experts of different disciplines. This multidisciplinary approach has gained ground over the past decades in modern infection units as a more effective treatment strategy, yielding better outcomes regarding infection eradication rates, length of hospital stay, and overall cost of treatments. Additionally, preventing and managing musculoskeletal infections requires strong connections between medical associations, biological laboratories, and the pharmaceutical industry worldwide. In this context, SICOT and World Association against Infection in Orthopaedics and Trauma (WAIOT) relationships have been increasing. The present editorial article discusses the multidisciplinary approach for managing bone and joint infections worldwide, explores the controversies in practices in terms of training, area of expertise, and extent of clinical involvement, and emphasizes the role of societies in research, prevention and management of musculoskeletal infections. The purpose is to acknowledge what orthopedics can obtain from specialists dealing with bone and joint infections and to consolidate their practice to provide the best care for orthopedic patients.

## The Impact of bone and joint infections

**Figure 1:**
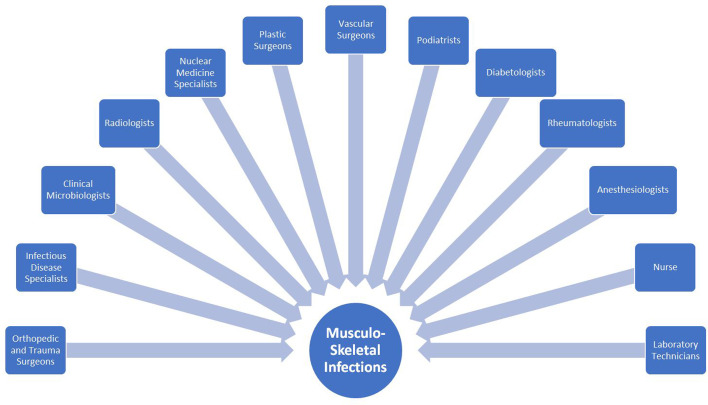
An (incomplete) list of healthcare professionals involved in managing musculoskeletal infections.

Bone and joint infections are associated with a devastating global burden, having an incidence rate of approximately 5/1,000 inhabitants in Europe [1]. These infections are responsible for 0.2% of all hospitalizations, while it is estimated that prosthetic joint infections account for 42% of all bone and joint infections [2, 3]. The collaboration of orthopedics with other medical specialties is paramount for managing patients with musculoskeletal infections. The successful treatment of these infections requires cooperation between orthopedic surgeons and experts of different disciplines (Figure 1). This multidisciplinary approach has gained ground over the past decades in modern infection units as a more effective treatment strategy, yielding better outcomes in terms of infection eradication rates, length of hospital stay, and overall cost of treatments [4, 5].

The present editorial article discusses the multidisciplinary approach for managing bone and joint infections worldwide, explores the controversies in practices in terms of training, area of expertise, and extent of clinical involvement, and emphasizes the role of societies in the research, prevention and management of musculoskeletal infections. The purpose is to acknowledge what orthopedics can obtain from specialists dealing with bone and joint infections and to consolidate their practice to provide the best care for orthopedic patients.

## Infectious disease specialists worldwide

Among the various specialists involved in bone and joint infection management, infectious disease specialists and clinical microbiologists are among the most involved in cooperating with an orthopedic surgeon in diagnostic and therapeutical pivotal decisions. It is estimated that bone and joint infections account for 7–30% of infectious disease consultations worldwide, highlighting the importance of infection specialists in managing patients with such infections [6–8]. Physicians of these two specialties, as members of the multidisciplinary team, can recommend the appropriate diagnostic tests such as antibiofilm pre-treatment of samples by sonication or by dithiothreitol, prolonged incubation of cultures if needed, molecular methods, serologic tests, and specific biomarkers depending on patients’ risk factors and clinical presentation. Moreover, they can recommend any additional investigation studies such as relevant imaging and interpret the results of these tests. Based on the results of these diagnostic tests, the available antibiotic options, and patients’ contraindications for specific antibiotics, infectious disease specialists can design the overall treatment plan and guide the antibiotic therapy in terms of selecting the appropriate antibiotic agents, recommend the overall duration of the antibiotic treatment, and advise on the timing of the transition from IV antibiotics to oral administration. The role of these physicians is even more highlighted in cases of complex bone and joint infections, unusual organisms, or in cases of multidrug-resistant microorganisms. Moreover, they can advise on specific intraoperative measures such as the antibiotic composition of the cement spacers and recommend other adjuncts to aid in eradicating the infection, such as hyperbaric oxygen therapy. Last, they can follow-up patients for any side effects of the antibiotic regime, ensure tolerability of the treatment, provide alternative options if necessary, and decide whether long-term antimicrobial suppressive therapy is needed.

However, although infection specialists and clinical microbiologists are nowadays an integral part of the health care workforce worldwide, there is a very diverse picture across the world in terms of training requirements, area of expertise, and extent of their clinical involvement in diagnosis and treatment of infectious diseases and particularly in the management of bone and joint infections.

In the United States (US), infectious disease physicians are required to complete a 2-year fellowship focused on the prevention, diagnosis, and treatment of diseases that are caused by microorganisms, such as bacteria, viruses, fungi, and parasites. Most infectious disease physicians are formally trained in internal medicine or pediatrics and decide to pursue a career in the field of infectious diseases following completion of their previous postgraduate residency training. Currently, there are 156 accredited infectious disease fellowship programs in the United States [9]. However, only one infectious disease fellowship program is dedicated solely to bone and joint infections (Mayo Clinic in Rochester, MN). In all other programs, management of bone and joint infections is part of their training curriculum. Clinical microbiology is another subspecialty in the United States in which physicians complete a 2-year fellowship program mainly focused on bench training in microbiology, usually after completing their residency program in pathology. Although bone and joint infections are part of the clinical microbiology fellowships and pathology residency programs, no independent training program is dedicated to bone and joint infections. Clinical microbiologists have a broad knowledge of clinical and diagnostic microbiology and are well qualified to direct microbiology laboratories while they are in close collaboration with infectious disease physicians. Although in many cases, there is an overlapping field of expertise between clinical microbiologists and infectious disease physicians, and both can be part of the multidisciplinary team for the management of bone and joint infections, clinical microbiologists are usually not directly involved in the clinical care of patients, whereas infectious disease physicians are being consulted to evaluate patients, admit patients, and care for their treatment.

In the United Kingdom (UK), infectious disease physicians receive slightly different training than those in the US. Overall, the training pathway to become an infectious disease physician in the UK takes 7 years, 2–3 years in internal medicine, and 4–5 years in medical microbiology [10]. Moreover, the density of infectious disease physicians in the UK is significantly lower than in the US; currently, there are approximately 130 infectious disease physicians working in the National Health Care System of England [11]. Medical or clinical (the terms are used interchangeably) microbiologists are also involved in treating bone and joint infections in the UK. Microbiologists complete a 4-year specialty training in microbiology and have the option to receive further training to become infectious disease physicians [12]. This is slightly different than the US, where postgraduate training in microbiology is part of the pathology residency programs, while in the UK, microbiology consists of an independent specialty with a dedicated 4-year postgraduate training program. There are approximately 490 consultant microbiologists in the UK [13]. Although, in some cases, microbiologists in the UK are responsible for the management of infections in hospitals, usually, these physicians are more focused on laboratory medicine and do not care for patients alone. Microbiologists most commonly provide clinical advice on the diagnosis/treatment of bone and joint infections if microbiology findings are unusual and need to be discussed with clinicians or if clinicians seek advice from microbiologists. Since a substantial part of the training is common for infectious disease physicians and microbiologists in UK, there is a broad-based workforce available for the management of bone and joint infections.

In France, following a central reform of the health system in 2008, a network of regional centers (CRIOAcs – Centres de Référence des Infections Ostéoarticulaires complexes) was founded where different specialties, including orthopedic surgeons, infectious disease physicians, microbiologists, and radiologists, are general practitioner coordinating their practice to provide health care to patients with complex bone and joint infections [14]. Moreover, in 2014 the national French Orthopedic Society (Société Francaise de Chirurgie Orthopédique et Traumatologique [SOFCOT] in collaboration with the national Infectious Disease Society (Société de Pathologie Infectieuse de Langue Française [SPILF]) created a postgraduate 2-year training program in which medical or surgical residents can be enrolled and receive a national diploma in the field of bone and joint infections. However, there is no formal training requirement in France for infectious disease physicians or microbiologists to be involved in managing bone and joint infections.

In Germany, infectious diseases management as a medical specialty has been slowly established, and the density of infectious disease physicians is limited compared to other countries like the United States and England. It is estimated that more than 1000 infectious disease physicians are needed in Germany, while there is a similar shortage of medical microbiologists in many areas [15]. Similar to other countries, clinical or medical (these terms are used interchangeably like in other European countries), microbiologists are more focused on laboratory medicine and bench working, while infectious disease physicians directly provide health care to patients. Although the importance of specialized bedside infection care is highlighted, the overlapping role of microbiologists and infectious disease physicians is more prominent in Germany, and the management of bone and joint infections consists of either infectious disease physicians or medical microbiologists, depending on the hospital/area.

In most other European countries, infectious disease physicians receive an additional 2-year training following completion of their residency program, while there is not any formal European training program focused on bone and joint infections. In Scandinavian countries like Sweden, there are infection clinics where infection specialists, along with orthopedic surgeons, can admit and treat patients with bone and joint infections [16]. In these clinics, orthopedic surgeons and infection specialists perform clinical visits, evaluate patients, and are responsible for the care of hospitalized patients. Moreover, microbiologists have a highly varying clinical/ laboratory role in Europe, while there is limited consistency in their training curriculums. Although microbiology was historically considered largely based on laboratory medicine, over the recent decade, microbiologists’ practice has evolved into a continuum between laboratory and clinically focused specialty, while in some countries, microbiologists can provide formal clinical consultations. In line with this, in many European countries, the multidisciplinary team for managing bone and joint infections consists of microbiologists or infectious disease physicians interchangeably, based on the availability of these two specialties [17, 18].

## WAIOT and the multidisciplinary management of bone and joint infections

The key role of the multidisciplinary approach in the management of bone and joint infections is well recognized by the World Association against Infection in Orthopaedics and Trauma (WAIOT), which is the first and currently, the largest scientific association focused on research, prevention, and management of musculoskeletal infection (MSI) and biofilm- and implant-related infections in Orthopaedics and Trauma. Founded in Vienna in 2017, WAIOT now counts more than 2200 specialists from all the disciplines involved in managing musculoskeletal infection, operating in more than 100 countries worldwide.

Among the main missions of WAIOT is to raise more widespread cooperation among all the specialists and health professionals to improve bone and joint infection management and to favor continuous education and training in the field. In order to accomplish this task, WAIOT is keen on bringing international experts together in an annual meeting to conduct a very rich scientific program that updates orthopedic trauma stakeholders with the most recent research and approaches in a matter of prevention, diagnosis, and management of musculoskeletal infections. The 1st WAIOT Congress was originally scheduled to be held in Thessaloniki, Greece; however, because of the COVID-19 pandemic, it was held virtual on August 28, 2021. As the first meeting of the society, it had substantial contributions from many countries that were considered satisfactory. The 2nd WAIOT Congress is to be held on September 1–2, 2022, in Grand Nile Tower Hotel, Cairo, Egypt, under the aegis of SICOT, the World Orthopedic Concern (WOC), the Limb Reconstruction Society, and AO Spine is expecting around 500 attendees from various disciplines and 500+ virtually who will be able to benefit from international CME accreditation provided by the Congress (https://www.waiot2022.com).

Preventing and managing musculoskeletal infections not only necessitate a strong and permanent cooperation between medical doctors of various disciplines, but also strong connections between medical associations, biological laboratories, and the pharmaceutical industry worldwide. For example, a successful partnership was born between SICOT and WAIOT to raise and improve awareness of this big musculoskeletal issue. Indeed, SICOT-WAIOT relationships have been increasing since the founding of the latter. Specifically, in:

- 2017: SICOT hosted during its World Congress in Cape Town the first WAIOT elective general assembly (GA). It also admitted in its program some important lectures on infections by WAIOT founding members.
- 2018: First WAIOT Symposium in the general program of SICOT and 2nd WAIOT GA in Montreal, Canada.
- 2019: 2nd WAIOT Symposium in the SICOT Congress program in Muscat, Oman, and 3rd WAIOT GA Moreover, we were highly pleased to see Prof Ashok Johari, immediate WAIOT Past President, becoming SICOT President.
- 2021: 3rd WAIOT Symposium in the SICOT Congress program in Budapest, Hungary.
- 2022: 4th WAIOT Symposium in the SICOT Congress program in Kuala Lumpur, Malaysia.

In order to consolidate links, all WAIOT symposia and SICOT Infection Sessions involved eminent speakers from both Societies. However, as a newborn and rising star, WAIOT relies on SICOT to play a better supportive role. This could be partially achieved by considering a concrete agreement of partnership. In addition, many WAIOT members are involved in more and more Congresses, Courses, Symposia, and Sessions such as the AAOT, AAOS, DKOU, LRS, AROM, IAC, EOA, ORTHOCON, TRAUMACON, SMACOT, etc. This common interest in musculoskeletal infection studies and management increases cooperation and is a good means for improving research and education as well as humanitarian objectives all over the world.

SICOT-J, the second academic journal of SICOT after International Orthopaedics, is growing. As a general scientific journal, it also publishes articles on bone and joint infection cases [19–23]. We aim to provide our readers with interesting and quality papers that are expected to be didactive and educative and maybe lead to a change in their practice.

## Epilogue

Bone and joint infections represent a silent epidemic that is causing every year thousands of deaths and disabilities throughout the world [24]. While multidisciplinary teams are a recognized plus in the prevention and management of musculoskeletal infection, their practical realization can be challenging and partial in many realities due to limited resources, logistical and practical issues, and a lack of standards in training and education. Specifically, the consistency of the multidisciplinary team can vary across the world regarding the exact specialty of the infection specialists. Generally, infectious disease physicians provide bedside care, and microbiologists are more focused on laboratory medicine; however, these lines are not always well defined, and the field of expertise of these two specialties can overlap depending on the country, health care system, and availability of the respective physicians.

Although the management of bone and joint infections is part of the training curriculum of most microbiology and infectious disease residency/fellowship programs worldwide, infectious disease physicians and microbiologists are not required to complete any independent training program to qualify as bone and joint infection specialists. In the United States, the multidisciplinary team for bone and joint infections is consisted of infectious disease physicians, while microbiologists have a very limited clinical role. Although this model is similar to that of the United Kingdom, the limited availability of infection disease physicians in all parts of the country and the largely common training between infection disease physicians and microbiologists results in a more variable model of multidisciplinary teams, in which the infection specialists of the team can be either infectious disease physicians or microbiologists. In other European countries, the interchangeable role of infectious disease physicians and microbiologists is even more prominent, and the multidisciplinary team can also consist of either one of these two specialties or even internal medicine physicians as the infection specialists of the team. A more unified training curriculum for the infectious disease physicians and microbiologists across the world, a more clear definition of their role, and the establishment of independent fellowship programs dedicated to bone and joint infections would aid in the optimization of the quality of the health care that is provided in patients with such infections.

In this panorama, multidisciplinary scientific associations such as WAIOT and SICOT may play an important role in raising awareness among all physicians dealing with musculoskeletal infections, as well as in increasing the opportunities for education and training in combined meetings with specialists from different disciplines in order to better standardize the procedures and increase the cooperation in multidisciplinary teams. The effects of such collaborations are expected to be faced on orthopedic training, practice, and science worldwide in the long-term.

## Notes

**Cite this article as:** Romanò CL, Tsantes AG, Papadopoulos DV, Tsuchiya H, Benzakour T, Benevenia J, del Sel H, Drago L, Mavrogenis AF & World Association against Infection in Orthopedics and Trauma (WAIOT) Study Group on Bone and Joint Infection Definitionsa (2022) Infectious disease specialists and teamwork strategies worldwide: the World Association against Infection in Orthopedics and Trauma (WAIOT) and SICOT continue to cooperate in fighting musculoskeletal infections. SICOT-J **8**, E1
